# Time course of estazolam in single-strand hair based on micro-segmental analysis after controlled oral administration

**DOI:** 10.3389/fchem.2022.996857

**Published:** 2022-10-17

**Authors:** Duoqi Xu, Jiaojiao Ji, Ping Xiang, Hui Yan, Gengli Duan, Min Shen

**Affiliations:** ^1^ Department of Forensic Toxicology, Academy of Forensic Science, Shanghai Key Laboratory of Forensic Medicine, Shanghai, China; ^2^ Department of Forensic Medicine of Shanghai Medical College, Fudan University, Shanghai, China; ^3^ Department of Phamaceutical Analysis, School of Phamacy, Fudan University, Shanghai, China

**Keywords:** drug incorporation, drug-facilitated sexual assault, estazolam, micro-segmental hair analysis, LC/MS-MS

## Abstract

The mechanism of estazolam incorporation into hair was investigated by studying the time course of estazolam along single-strand hair after two oral administration of estazolam at 28 days interval. Estazolam in single hair segments 0.4 mm in length was verified and quantified by ultra-high-performance liquid chromatography coupled to tandem mass spectrometry (UPLC-MS/MS). The distributions of estazolam within a strand of hair (collected at 12 h, 28 days, and 56 days post-administration) were visualized by micro-segmental analysis. The highest estazolam concentration (1.5–9.9 pg/mm) was detected in the hair bulb region (S1), and it then decreased through the hair shaft to the distal end, with a small fluctuation (0.3–3 pg/mm) near the junction of the hair roots and shafts (S4–S7) 12 h after drug intake. These findings suggested that the incorporation of estazolam occurred in two regions, mainly in the hair bulb and to a lesser extent in the upper dermis zone. Models using internal temporal markers (TIMs) and temporal intervals (TIs) were constructed to estimate the day of estazolam ingestion. The estimation accuracy was within an average error of 1.7 mm and 3.0 mm between the calculated and actual positions, based on the TIMs and TIs 56 days after estazolam intake. These findings can help in further elucidation of the drug incorporation mechanism, which is crucial for interpreting hair analysis results used to reveal individual drug-use history.

## 1 Introduction

Since the late 1990s, hair testing for drugs in humans has been increasingly applied in the forensics, clinical, and anti-doping fields (Chèze et al., 2005; Xiang et al., 2011; Franois et al., 2014; Salomone et al., 2017; Wang et al., 2019). In forensic toxicology, hair testing has been used to provide drug exposure history in drug-related deaths, drug-facilitated crimes (DFCs), child protection (Scott, 2009; De Castro et al., 2012; Alvarez et al., 2018), and for monitoring drug misuse in drug rehabilitation programs and workplace drug testing (Salomone et al., 2016; Leung et al., 2018; Stowe et al., 2019). When compared with blood testing, hair analysis has some advantages, such as non-invasive sample collection, relatively easy performance, long detection window of drugs over months to years, and ability for use under close supervision of law enforcement officers in forensic situations to prevent adulteration or substitution (Nakahara et al., 1992; Kintz, 2017).

**FIGURE 1:**
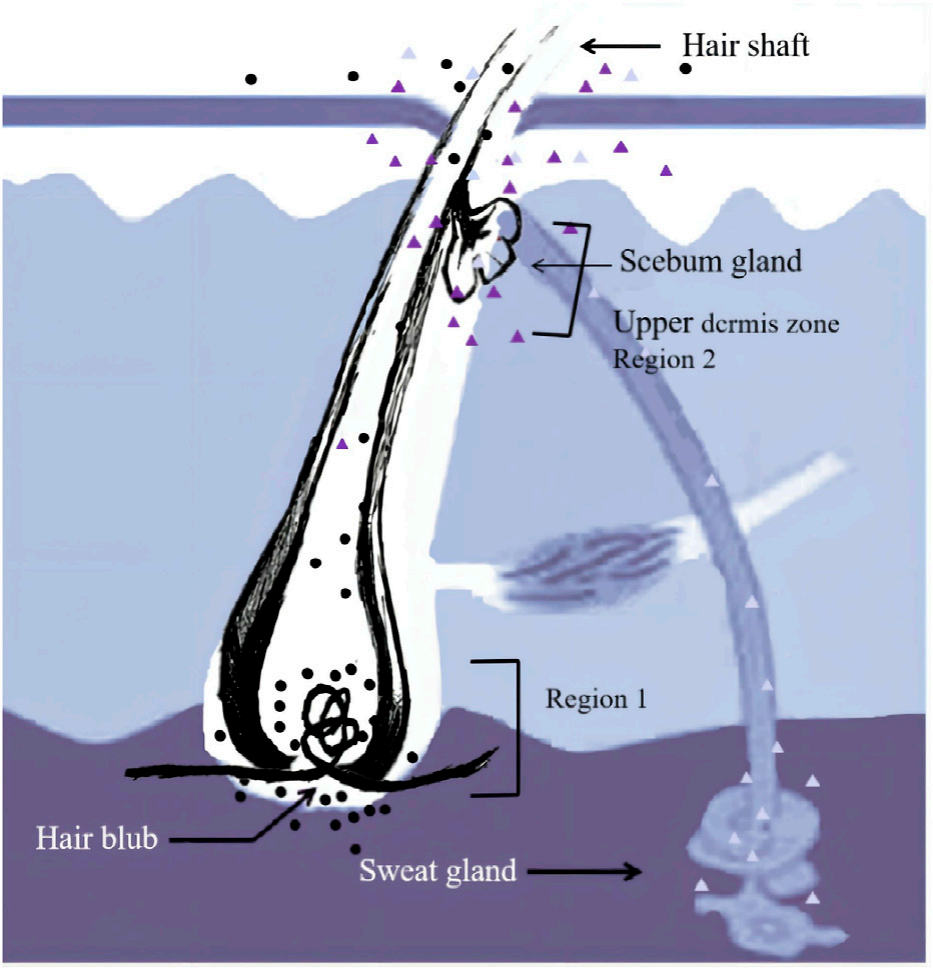
Incorporation routes into the hair. Region 1 and 2 correspond to the hair bulb and the upper dermis zone, respectively.

The incorporation pathways of drugs into hair have been clarified by several researchers who have analyzed substances/metabolites in plucked hairs after single doses of a drug/substance of interest (Miki et al., 2011; Schräder et al., 2012; Kamata et al., 2015; Shima et al., 2017; Shima et al., 2019; Kamata et al., 2020; Nitta et al., 2021). For example, Shima et al. (Shima et al., 2017) revealed that zolpidem is incorporated into two regions of the hair root (Figure 1). Zolpidem in Region 1 was absorbed from the hair bulb via the bloodstream and distributed over the whole hair root, whereas zolpidem in the distal region (Region 2) was probably incorporated into the hairs through sweat and/or sebum that soaked the hair root near the scalp surface. A comparison of the incorporation of methoxyphenamine and zolpidem into white and black hairs by Shima et al. (Shima et al., 2019) revealed abundant drugs in the black hairs in 0–1 mm segments that included the hair bulb (Region 1), while the white hairs showed no or trace levels of these drugs in the 0–1 mm segments at 12–36 h after drug intake. Their findings suggest that hair pigments have two important roles in the distribution of drugs: one involving the incorporation of drugs into hair via Region 1 and the other involving the retention of drugs already incorporated.

Micro-segmental analysis, which was developed by Kuwayama et al. (Kuwayama et al., 2016; Kuwayama et al., 2017; Kuwayama et al., 2018a; Kuwayama et al., 2018b; Kuwayama et al., 2018c; Kuwayama et al., 2019a), involves the segmentation of an individual hair strand at 0.4 mm intervals, a length that corresponds to the average daily hair growth (Kuwayama et al., 2018a). Using this method, a drug ingested in a single dose can be located in a specific region of a hair strand several millimeters in length, thereby pinpointing the day of ingestion (Kuwayama et al., 2018a). This micro-segmental hair analysis method was capable of estimating the day of drug ingestion in DFSA (Drug-facilitated Sexual Assault, DFSA) or other forensic cases involving drug intake (Kuwayama et al., 2018b; Kuwayama et al., 2019a; Kuwayama et al., 2019b; Wiedfeld et al., 2021; Xu et al., 2022).

Many models, such as those utilizing internal temporal markers (ITMs), have been used to estimate the day of drug administration (Kuwayama et al., 2018c). The compounds ingested on two known days are used as ITMs and are detected at specific positions in the analyzed hairs. The rate of hair growth can then be calculated from the distance between the detection of the indicator compounds and the time intervals between the administrations of the drug compounds. The day of drug ingestion is then estimated from the calculated rate of hair growth and the position of the drug peak maximum. The estimation accuracy was within an average error of 2 days (Kuwayama et al., 2018c). This method was applied by Wiedfeld (Wiedfeld et al., 2021) by collecting two hair samples within a defined timeframe after the incident. Calculation of the distance between the position of the mean peak maximum in the first and second samples and dividing it by the time interval between samplings gave the individual mean growth rate and revealed a gap of 5–6 mm between the calculated and actual positions. During an investigation of an unnatural death case, the day of death was estimated using the known surgery day, the distance from the hair root to the lidocaine peak in the hair strand, and the average hair growth rate (0.4 mm/day). The day of death estimated using hair analysis (52.9 ± 3.2 days after the surgery day) corresponded to approximately 4 days before the corpse was discovered (Kuwayama et al., 2019b). Midazolam was successfully detected in proximal 5.6–6.8 mm segments by micro-segmental hair analysis in three hair strands collected 16 days after the event, whereas it had not been detected in the proximal 3 cm by conventional segmental hair analysis (Xu et al., 2022).

One drug of particular interest in forensic hair analysis cases is estazolam (Ramírez Fernández et al., 2015; Kasai et al., 2019; Sha et al., 2020), a prescription sedation and anesthetic that is one of the most frequently encountered prescription benzodiazepines in cases of drug-facilitated sexual assault (DFSA) (Xiang et al., 2011) Estazolam was detected in the femoral muscle (39.9 ng/g) and bone marrow of a cadaver that had transformed into adipocere in wet and cold conditions (Kasai et al., 2019). Meanwhile, hair collected and analyzed from 14 volunteers 1 month after estazolam administration (1–6 mg) was positive for estazolam in all the proximal 0–2 cm segments (Xiang et al., 2011), suggesting that hair analysis could be beneficial in determining time of death in difficult forensic cases. With increased dosage, estazolam can be detected in 2–4 cm segments and even in some 4–6 cm segments.

The aim of the present study was to investigate use LC-MS/MS and micro-segmental hair analysis to determine the incorporation sites of estazolam in hair. The time course of estazolam incorporation was evaluated in 0.4 mm hair segments along the hair shaft using hair plucked 12 h, 28 days, and 56 days after two oral administrations of estazolam at 0 days and 28 days.

## 2 Materials and methods

### 2.1 Chemicals and reagents

Estazolam, and the internal standard (IS) diazepam-d_5_ standard stock solutions (1 mg/ml) were purchased from Cerilliant (Round Rock, TX, United States). Methanol, and acetonitrile (≥99.9%, suitable for UPLC) were purchased from Sigma-Aldrich (St. Louis, MO, United States). Ammonium formate and formic acid were obtained from Fluka (Buchs, Switzerland). Dithiothreitol (DTT) powder (≥99%, HPLC-grade) was obtained from Sigma-Aldrich (St. Louis, MO, United States). Deionized water was purified using a Milli-Q system (Millipore, MA, United States). Custom-made transparent stationery tape ruled in 0.4 mm squares was purchased from Qian Jie Metal Label Factory (Shanghai, China).

Working solutions of estazolam (2000, 1000, 400, 100, 50, 20, and 10 pg/ml) were diluted in methanol from a stock solution and stored at -20°C. The hair extraction medium (EM) was prepared by dissolving DTT in a mixture of methanol/acetonitrile/2 mM ammonium formate (8% acetonitrile, pH 5.3) (25:25:50, v/v/v) at a concentration of 10 mg/ml. The diazepam-d_5_ (internal standard, IS) working solution (10 pg/ml) was diluted with EM solution.

### 2.2 Micro-segmental hair preparation

An individual strand of hair was wiped three times with methanol, extended along the ruled tape, and attached to a foam board. The hair was segmented with surgical scissors under an illuminated magnifying glass (30×), dividing the proximal 1 cm hair segment (root) into 25 segments (S1, S2, S3…. S25) 0.4 mm in length. Each segment was placed in a 200 µL tube, and 25 µL IS solution was added. After ultrasonication for 1 h and incubation for 20 h, the supernatant was transferred to an autosampler vial, and 10 µL was injected into the UPLC-MS/MS system.

### 2.3 Instrument

Chromatography was performed on a Sciex Exion UPLC system (AB Sciex, Foster City, United States) equipped with a Kinetex F5 column (100 × 2.1 mm, 2.6 μm i.d., Phenomenex, United States). A gradient elution was performed using 0.05% formic acid in water (mobile phase A) and acetonitrile (mobile phase B). The LC mobile phase gradient elution program is shown in Table 1. The autosampler was set at 4°C.

**TABLE 1:** LC mobile phase gradient elution program.

Time (min)	Flow (mL/min)	A%	B%
Initial	0.55	80.0	20.0
1	0.55	55.0	45.0
3.80	0.55	55.0	45.0
4.80	0.55	5.0	95.0
5.80	0.55	5.0	95.0
6.70	0.55	80.0	20.0
6.80	0.55	80.0	20.0
8.00	0.55	80.0	20.0

An AB Sciex 7500 Qtrap ™ triple quadrupole mass spectrometer (AB Sciex, Foster City, United States) was used in the positive electrospray ionization (ESI^+^) mode with a multiple reaction monitoring (MRM) mode. The optimum conditions for estazolam analysis were as follows: ion spray voltage (ISV), 1600 V; source temperature (TEM), 750°C; curtain gas (CUR), 36 psi; nebulizing gas (GS1), 40 psi; and heater gas (GS2), 70 psi. The precursor ions, product ions, collision energy (CE) values, and retention times for estazolam and the IS are shown in Table 2.

**TABLE 2:** MRM transitions and mass parameters of compounds.

Compounds	Precusor ion (m/z)	Product ion (m/z)	CE (eV)	RT (min)
Estazolam	295.1	**205.1** ^a^	37	3.85
		267.1	37	
IS	290.2	**198.2**	46	4.61
		154.4	40	

^a^Product Ion represented in bold were used for quantification.

### 2.4 Method validation

**FIGURE 2:**
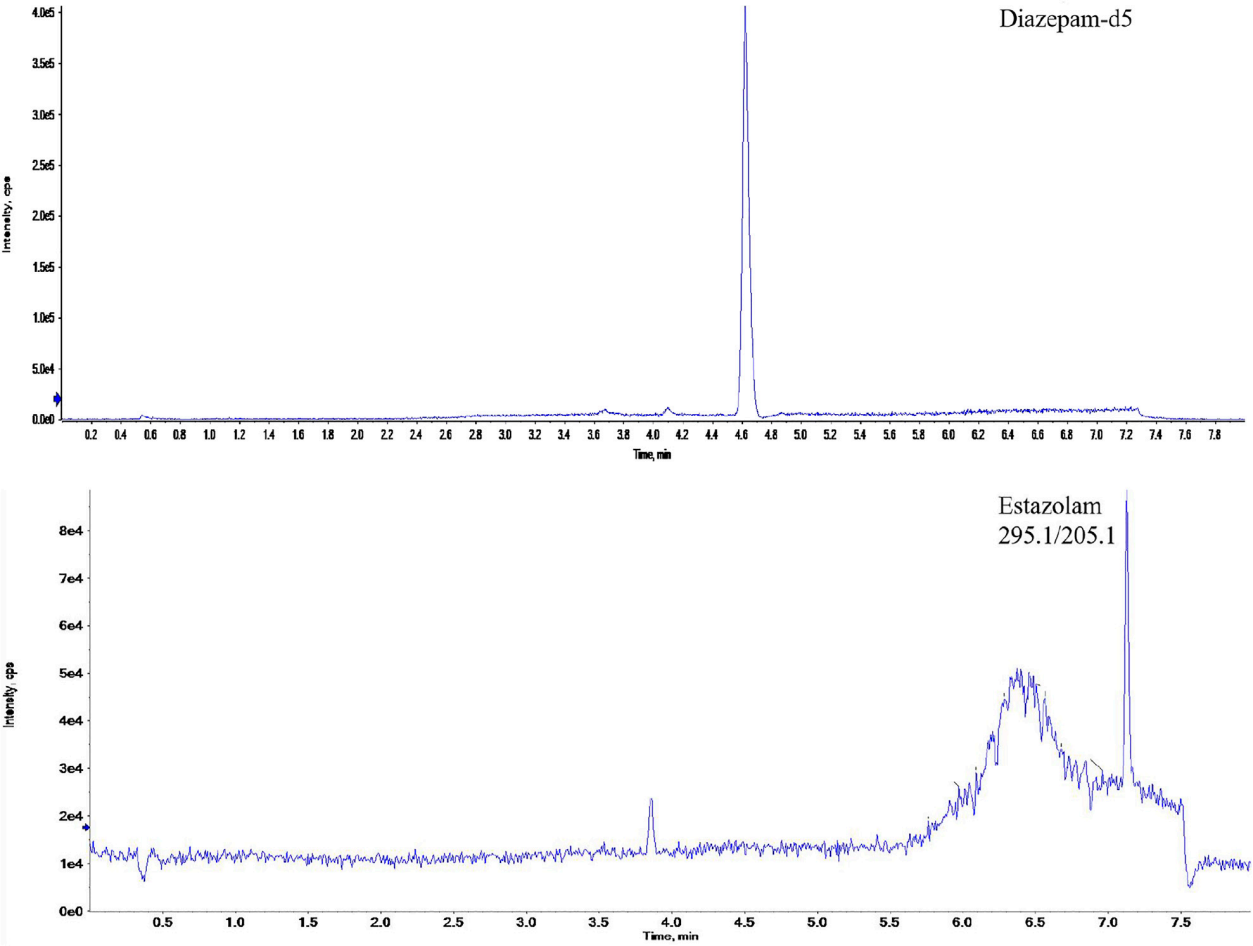
The ion chromatograms of a real hair sample with estazolam and the internal standard (diazepam-d5).

Drug-free hair samples from healthy volunteers were analyzed to ensure that no endogenous interfering peaks were present at the retention times of the analytes. Assay selectivity was confirmed by the absence of interfering peaks at the retention times for estazolam in blank hair. Working solutions of estazolam were spiked to blank hair to get spiked hair samples with concentrations at 0.05, 0.1, 0.25, 0.5, 2, 5, and 10 pg/mm (n = 3). Good linearity was observed in the range of 0.05–10 pg/mm for estazolam (Y = 0.47X-0.016), and *r* ^2^ = 0.9992. The LOD and LLOQ were determined using spiked blank 0.4 mm hair samples covering a estazolam range from 0.01 to 0.05 pg/mm. Limit of detection (LOD) for estazolam was determined by decreasing their concentrations until a reproducible instrument response greater than or equal to three times the background noise was observed. Lower limit of quantification (LLOQ) was defined as the lowest concentration in the calibration curve, at which the signal of analyte was greater than ten times the baseline. The LOD and LLOQ for estazolam in hair were 0.03 pg/mm and 0.05 pg/mm, respectively. The chromatograms of a hair sample from subject 1# is shown in Figure 2.

### 2.5 Sample collection

Four healthy volunteers (Subjects ^#^1–^#^4, one female and three males with straight black hair, ages 25–31 years) who had not come into contact with estazolam in the past 6 months were recruited into the study. All subjects agreed to participate in the experiment through written consent. This study was approved by the Ethics Committee for research on human subjects at Academy of Forensic Science, China.

All four subjects ingested 1 mg estazolam at 0 days and 28 days (after initial hair sampling). Hair samples (n = 5) were plucked from the posterior vertex region using tweezers at 12 h, 28 days, and 56 days. Micro-segmental analysis was carried out on the proximal 4.8 mm (S1–S12) segment (root) of the hair samples plucked 12 h after intake, the proximal 2 cm (S1–S50) segment (root) of hair samples plucked 28 days post-administration and the proximal 4 cm (S1–S100) segment (root) of hair samples plucked 56 days post-administration.

## 3 Results

### 3.1 Distribution of estazolam in hair roots 12 h after administration

Estazolam was detected in almost all hair segments (S1–S12) in the hair root samples (n = 5), including the hair bulb, from Subject^#^1–Subject^#^4 collected 12 h after a single dose, as shown in Tables 3-6. Estazolam was most abundantly distributed in the 0–0.4 mm segments (S1) at concentrations ranging from 1.5 to 9.9 pg/mm single hair. A small fluctuation was observed in the upper part of the hair root in most hairs, corresponding to the junction of the hair roots and shafts (S4–S7, 0.3–3 pg/mm). Trace amounts of estazolam were also detected in 3.2–4.8 mm segments (S9–S12), which correspond to the hair shaft outside the scalp surface.

**TABLE 3:** Concentration of estazolam in hair micro-segments of Subject^#^1 plucked at 12 h after estazolam intake (n = 5).

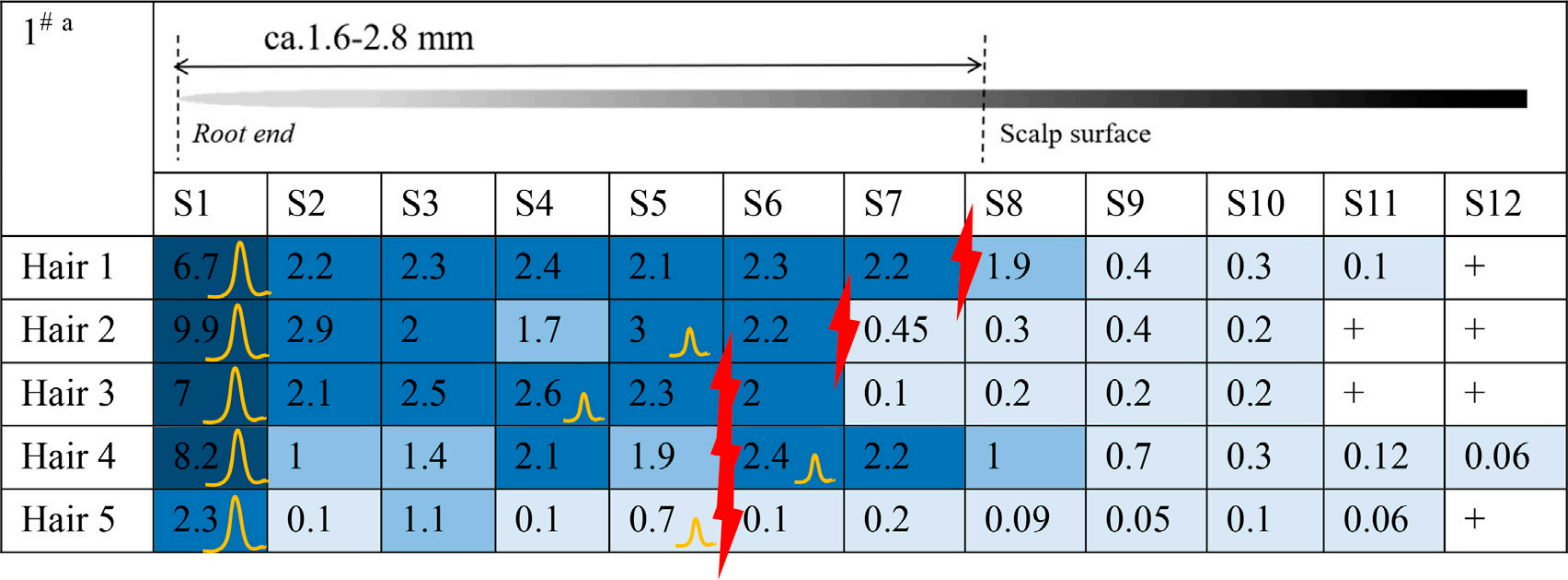

a Different colors illustrated different ranges of estazolam: 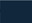
More than 5 pg/mm, 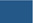
2–5 pg/mm, 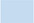
1–2 pg/mm, 0.05–1 pg/mm,+, 0.03–0.05 pg/mm, -, undetected.

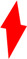
the junction of the hair roots and shafts.

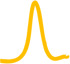
The highest peak.

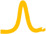
The second peak.

**TABLE 4:** Concentration of estazolam in hair micro-segments of Subject^#^2 plucked at 12 h after estazolam intake (n = 5).

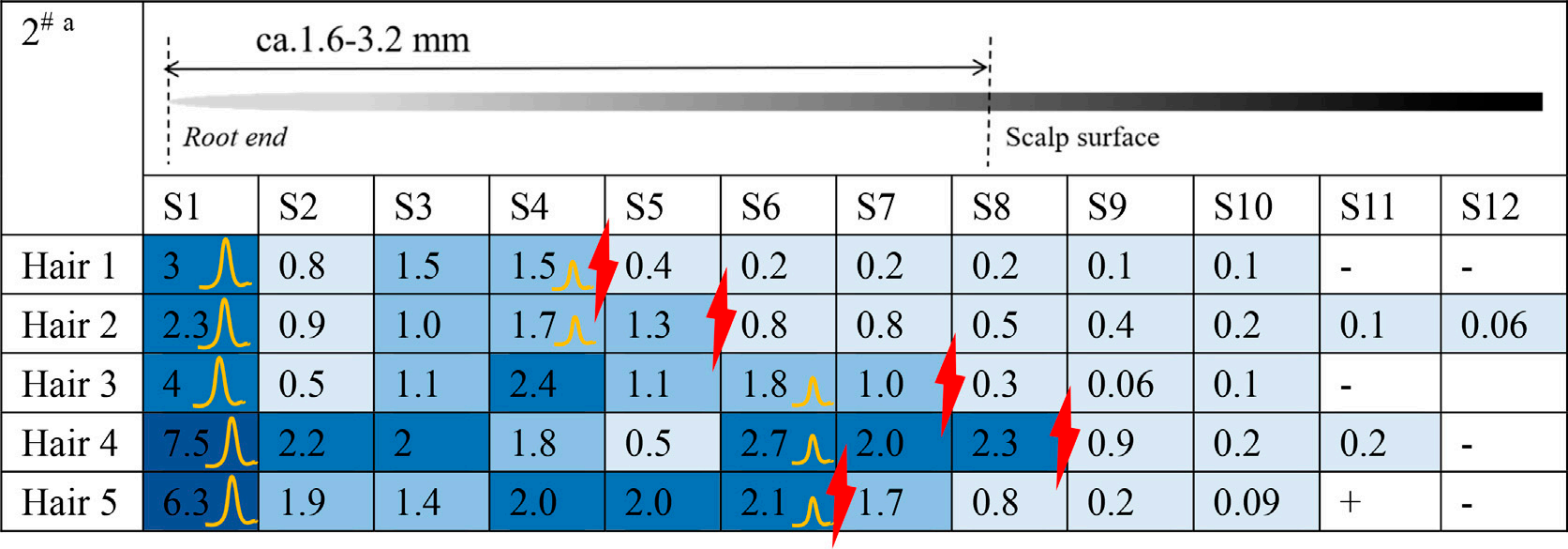

a Different colors illustrated different ranges of estazolam: 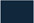
More than 5 pg/mm, 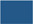
2–5 pg/mm, 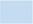
1–2 pg/mm, 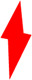
0.05–1 pg/mm, +, 0.03–0.05 pg/mm, -, undetected.

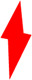
The junction of the hair roots and shafts.

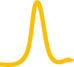
The junction of the hair roots and shafts.

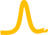
The highest peak.

**TABLE 5:** Concentration of estazolam in hair micro-segments of Subject^#^3 plucked at 12 h after estazolam intake (n = 5).

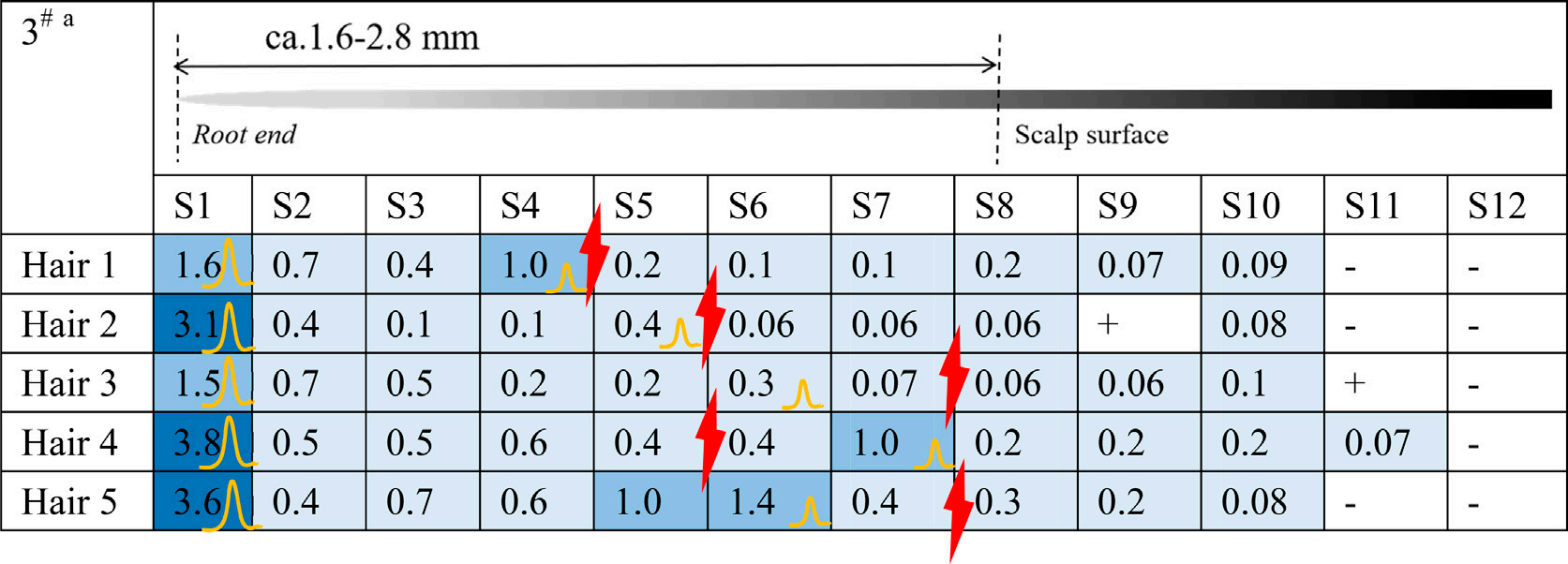

a Different colors illustrated different ranges of estazolam: 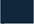
More than 5 pg/mm, 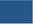
2–5 pg/mm, 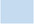
1–2 pg/mm, 0.05–1 pg/mm, +, 0.03–0.05 pg/mm, -, undetected.

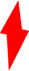
The junction of the hair roots and shafts.

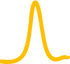
The highest peak.

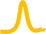
The second peak.

**TABLE 6:** Concentration of estazolam in hair micro-segments of Subject^#^4 plucked at 12 h after estazolam intake (n = 5).

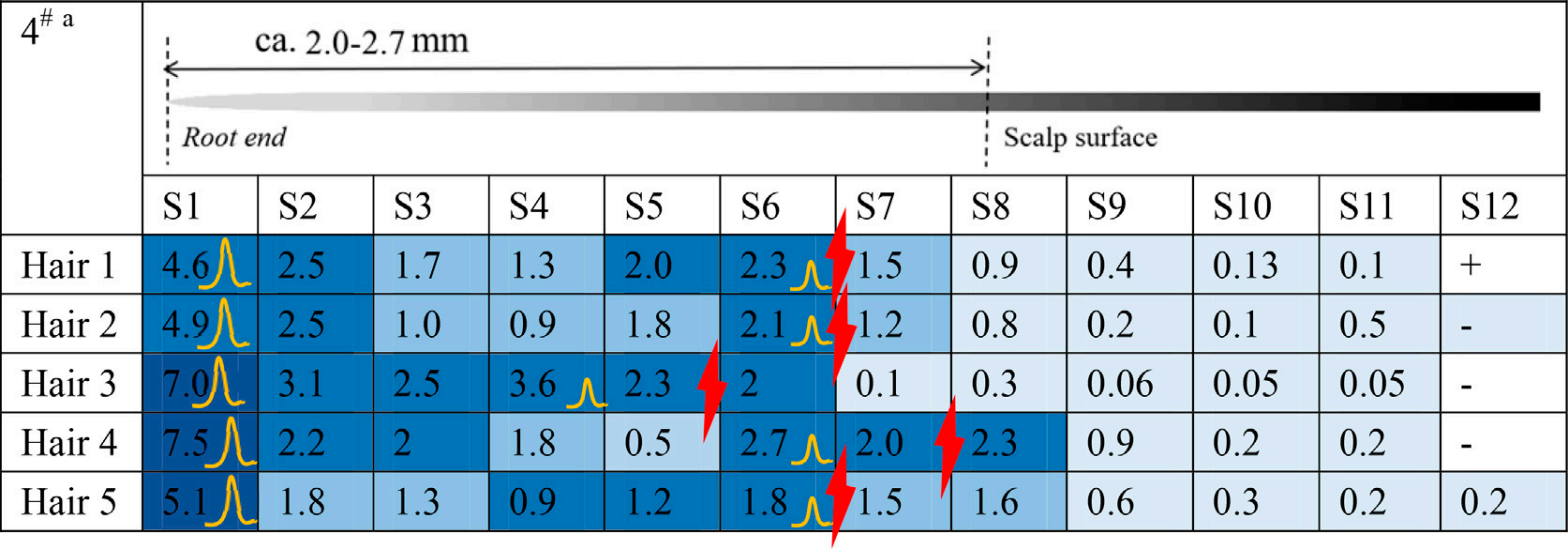

^a^Different colors illustrated different ranges of estazolam: 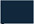
More than 5 pg/mm, 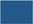
2–5 pg/mm, 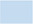
1–2 pg/mm, 0.05–1 pg/mm, +, 0.03–0.05 pg/mm, -, undetected.

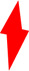
The junction of the hair roots and shafts.

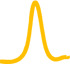
The highest peak.

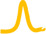
The second peak.

### 3.2 Distribution of estazolam along hair strands plucked at 28 days and 56 days

**FIGURE 3:**
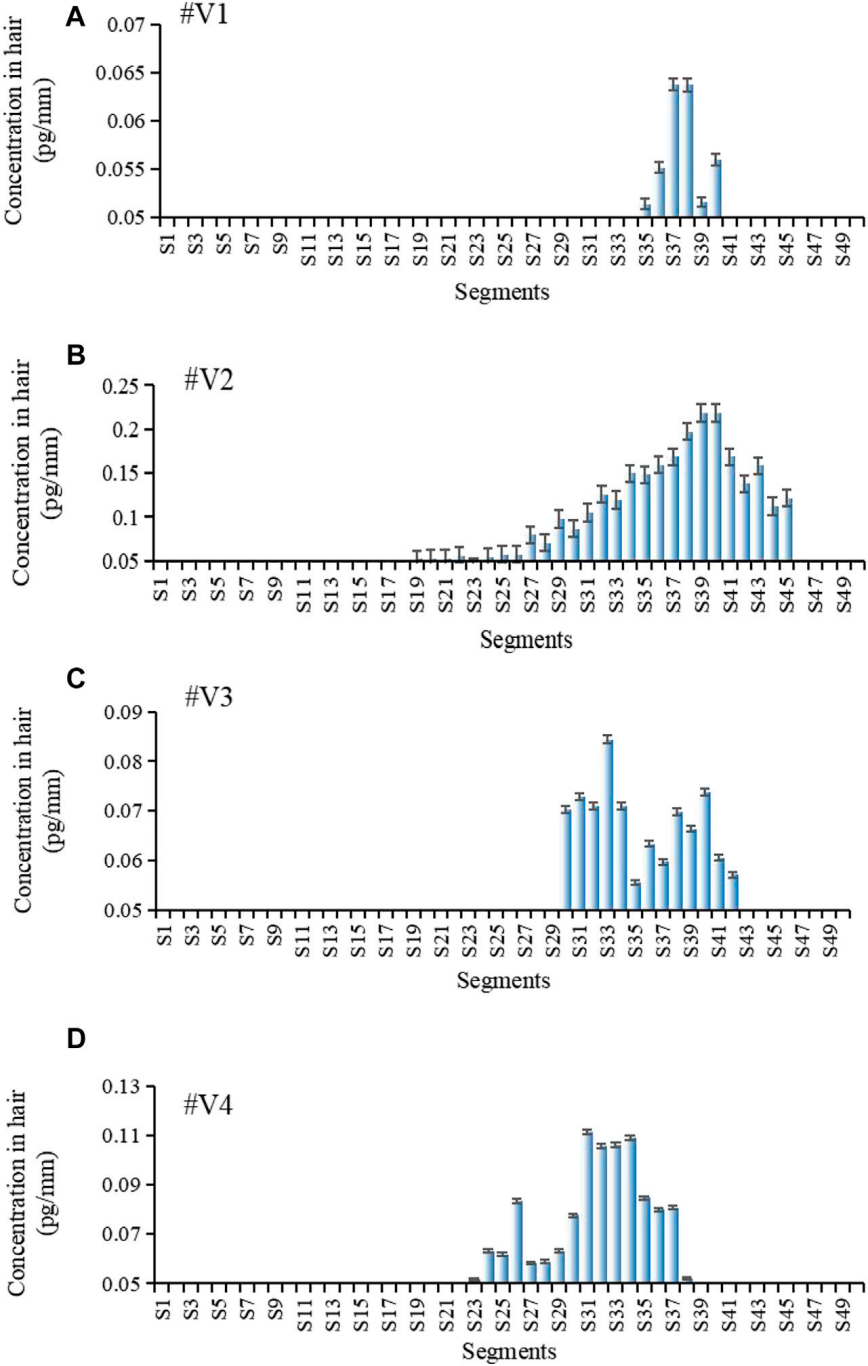
Estazolam concentration in each 0.4 mm segment along single-strand hair collected by plucking from Subject #1-#4 28 d after estazolam intake. Estazolam concentration in each 0.4 mm segment along single-strand hair collected by plucking from Subject #1–#4 [**(A)**: Subject #1, **(B)**: Subject #2, **(C)**: Subject #3, **(D)**: Subject 4] 28 d after estazolam intake.

Figure 3 shows the estazolam concentration in hair samples plucked at 28 days from Subjects^#^1–^#^4. Estazolam (0.05–0.65 pg/mm) was detected in proximal 7.2–18 mm segments (S19–S45), but not in the S1–S18 and S46–S50 segments, and the concentration peak appeared in the proximal 11.6–16.8 mm segments (S30–S42) (Table 7).

**TABLE 7:** Distribution of estazolam along hair strands after 28 days dose (n = 5).

Voluntaries	Samples	Peak1	Voluntaries	Samples	Peak1
^#^V1	No.1	S38	^#^V3	No.1	S38
	No.2	S38		No.2	S36
	No.3	S31		No.3	S40
	No.4	S33		No.4	S42
	No.5	S42		No.5	S38
^#^V2	No.1	S40	^#^V4	No.1	S33
	No.2	S39		No.2	S30
	No.3	S36		No.3	S34
	No.4	S39		No.4	S32
	No.5	S38		No.5	S34

**FIGURE 4:**
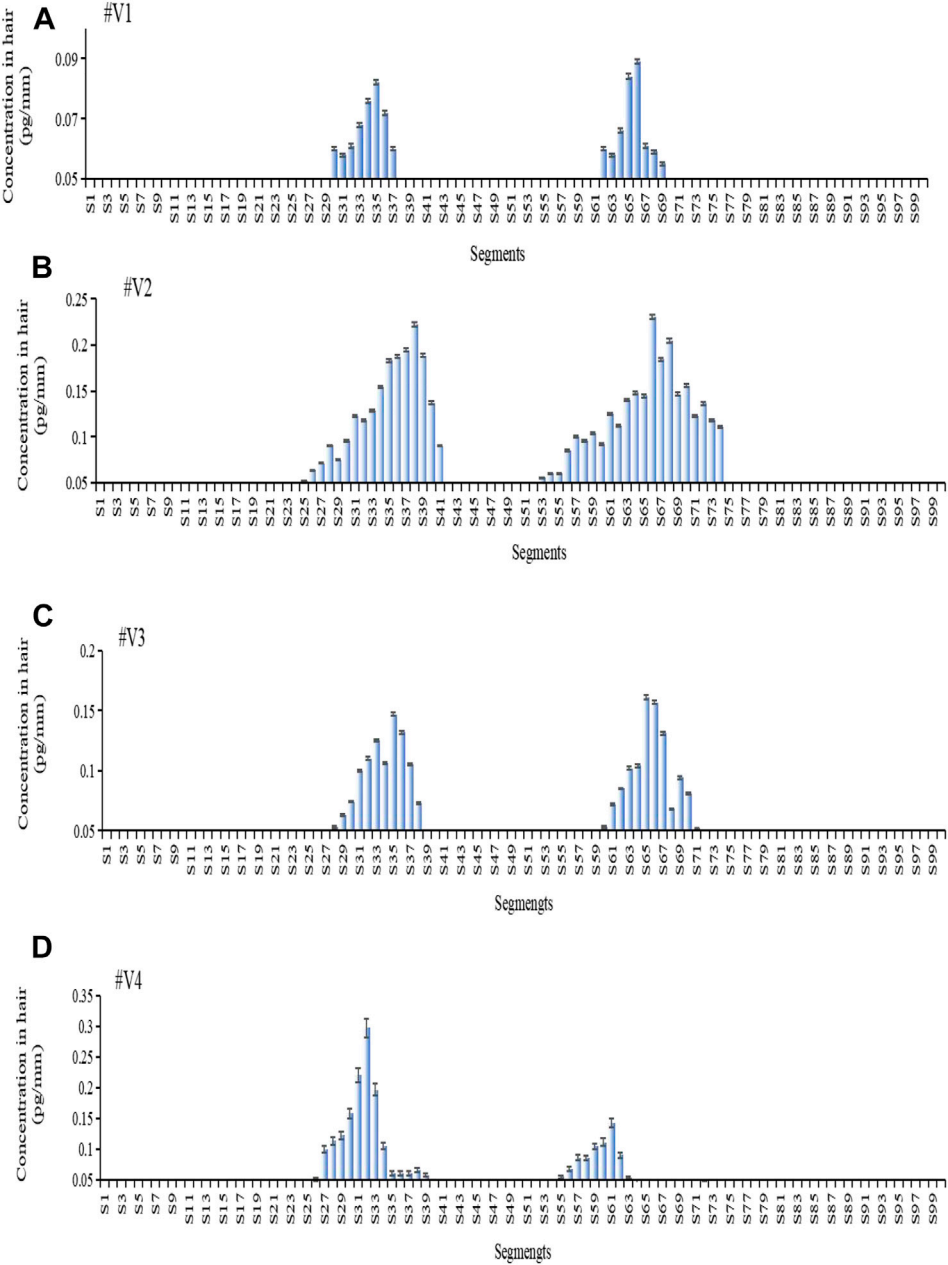
Estazolam concentration in each 0.4 mm segment along single-strand hair collected by plucking from Subject ^#^1- ^#^4 56 days after estazolam intake. Estazolam concentration in each 0.4 mm segment along single-strand hair collected by plucking from Subject #1–#4 [**(A)**: Subject #1, **(B)**: Subject #2, **(C)**: Subject #3, **(D)**: Subject 4] 56 d after estazolam intake.

Figure 4 shows the estazolam concentration in hair samples plucked at 56 days (28 days after the second oral dose) from Subjects^#^1–^#^4. Estazolam (0.05–0.29 pg/mm) was detected in the proximal 9.6–16.4 mm segments (S25–S41), and in the proximal 20.8–29.6 mm segments (S53–S74), but not in the other segments (S1–S24, S75–S100). Two peaks of estazolam appeared at the proximal 11.6–15.6 mm (S30–S39), and the proximal 23.2–28 mm (S59–S70) of the hair, corresponding to the two administrations of estazolam (Table 8).

**TABLE 8:** Distribution of estazolam along hair strands after 56 days dose (n = 5).

Voluntaries	Samples	Peak Maxima (Peak2)	Peak Maxima (Peak3)
^#^V1	No.1	S66	S30
	No.2	S65	S34
	No.3	S76	S39
	No.4	S73	S38
	No.5	S67	S36
^#^V2	No.1	S67	S38
	No.2	S66	S35
	No.3	S68	S39
	No.4	S64	S34
	No.5	S59	S32
^#^V3	No.1	S67	S35
	No.2	S68	S31
	No.3	S66	S36
	No.4	S68	S38
	No.5	S65	S37
^#^V4	No.1	S59	S30
	No.2	S62	S32
	No.3	S55	S30
	No.4	S61	S32
	No.5	S61	S30

## 4 Discussion

### 4.1 Incorporation pathways of estazolam into hair

The time course of estazolam incorporation in the hair root in the short timeframe (i.e., 24 h) following intake would be key in unveiling the mechanism of its incorporation into hair (Nitta et al., 2021). At 12 h after intake, the highest amounts of estazolam were localized in the 0–0.4 mm segment (S1) that included the hair bulb, along with a small fluctuation that appeared at the junction of the hair root and shaft (S4–S7). These findings indicated the occurrence of two major drug incorporation sites: the hair bulb and the upper dermis zone of the hair root. Two drug peaks in hair were also reported in other studies for zolpidem and methoxyphenamine after a single dose (Shima et al., 2017; Nitta et al., 2021).

#### 4.1.1 Estazolam incorporation into the hair bulb

In this study, estazolam in the 0–0.4 mm segment was most abundant at 12 h. Plasma estazolam concentrations peaked at approximately 1.5 h after a single oral dose (n = 14) and then declined at a slow rate for several hours (Xiang et al., 2011). Estazolam took a longer time to achieve a peak level in the hair bulb than in the plasma, suggesting that estazolam was gradually accumulated in the hair bulb region after a single administration. Similar findings have been reported for methoxyphenamine, which showed rapid increases in plasma that reached maximum concentrations 1 h after intake but only became detectable in hair 30 min after intake in the hair bulb (0–1 mm) and 1 h after intake in the upper dermis zone (2–4 mm) (Nitta et al., 2021).

#### 4.1.2 Drug incorporation into the upper dermis zone

The small peak appearing in the tip side of the hair roots and shafts (S4–S7) indicates a drug incorporation site in the upper dermis zone of the hair root. Estazolam is incorporated into the hairs, probably through sweat and/or sebum that soaks the hair root near the scalp surface. The site of the small peaks depended on the length of hair roots, and most were located in the upper zone of the hair root, below the junction between hair root and hair shaft.

Estazolam is incorporated mainly through Region 1. However, previous studies have suggested that the mechanisms of drug uptake into hair and the main incorporation pathways differ significantly depending on the properties of each drug (Kuwayama et al., 2018a). Highly lipophilic compounds, such as zolpidem, are incorporated mainly through Region 1, whereas the sweat/sebum route might be more dominant than the blood route for the uptake of highly polar compounds, such as ethyl glucuronide, into hair (Schräder et al., 2012). Compounds with average basicity and lipophilicity, such as methoxyphenamine, would be incorporated into both regions at comparable levels (Nitta et al., 2021).

### 4.2 Estimation of the day of drug intake

Assuming that incorporation via the bloodstream is the main route for most drugs into the hair, the influence of blood circulation on the translational speed of the highest concentration segments must be considered when calculating the time of administration. Kuwayama (Kuwayama et al., 2018c) indicated a need to obtain hair samples for testing approximately 1 month after drug intake.

#### 4.2.1 The temporal interval (TI) model

The TIs are used to estimate the day of drug intake based on two hair samplings at $T_{1}$ (28 days) and $T_{2}$ (56 days), with no additional drug ingestion. $S_{T_{1}}$ and $S_{T_{2}}$ represent the hair segment numbers that correspond to the peak maxima of the drug collected at T_1_ and T_2_. The individual hair growth rate (V) was calculated using Eq. 1, based on the temporal intervals ( ΔT ) between $T_{1}$ and $T_{2}$ , and the distance ( ΔS ) between $S_{T_{1}}$ and $S_{T_{2}}$ _._

$$V=\frac{\text{Δ}S\cdot 0.4}{\text{Δ}T}=\frac{\left(S_{T_{2}}-S_{T_{1}}\right)\cdot 0.4}{T_{2}-T_{1}}$$ (1)

**FIGURE 5:**
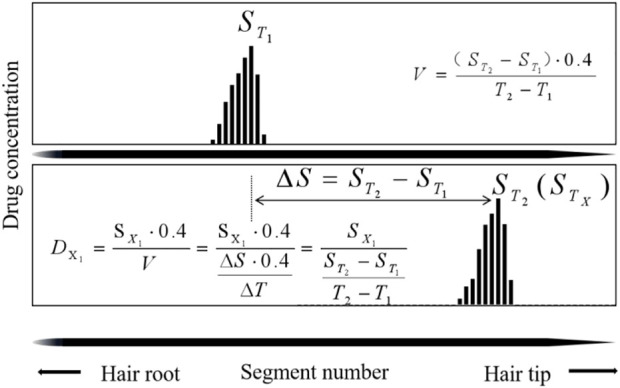
Calculation methods to estimate drug intake day ( $D_{X_{1}}$ ) by Temporal Intervals.

The first sampling day from the day of ingestion ( $D_{X_{1}}$ ) was estimated based on the formula of Eq. 2, with $S_{X_{1}}$ representing the segment number that corresponds to the peak maxima of estazolam in the hair collected at T_1_ (Figure 5).

$$D_{{\text{X}}_{1}}=\frac{{\text{S}}_{X_{1}}\cdot 0.4}{V}=\frac{{\text{S}}_{{\text{X}}_{1}}\cdot 0.4}{\frac{\text{Δ}S\cdot 0.4}{\text{Δ}T}}=\frac{S_{X_{1}}}{\frac{S_{T_{2}}-S_{T_{1}}}{T_{2}-T_{1}}}$$ (2)

The first sampling time interval from the day of ingestion was estimated by TIs in Table 9. The deviations, which ranged from 0.1 to 14.1 d (average 7.5 ± 3.9 days, n = 20) between the calculated ( $D_{X_{1}}$ ) and actual day (28 days) calculated by “ $D_{X_{1}}$ - 28 days,” included 10% within 2 days, 20% from 2 to 5 days, 40% from 5 to 10 days, and 10% beyond 10 days.

$$D_{{\text{X}}_{2}}=\frac{{\text{S}}_{X_{2}}\cdot 0.4}{V}=\frac{{\text{S}}_{{\text{X}}_{2}}\cdot 0.4}{\frac{\text{Δ}S\cdot 0.4}{\text{Δ}T}}=\frac{S_{X_{2}}}{\frac{S_{T_{2}}-S_{T_{1}}}{T_{2}-T_{1}}}$$ (3)

**TABLE 9:** The estimation using TIs.

Voluntaries	Samples	Rate (mm/day)	Dx_1_ (Day)	Deviation		Dx_2_ (Day)	Deviation	
				(Day)	(mm)		(Day)	(mm)
^#^V1	No.1	0.47	32.3	4.3	1.7	56.0	0.2	0.08
	No.2		32.3	4.3	1.7	55.2	0.7	0.08
	No.3		26.4	1.6	0.6	64.5	8.7	0.3
	No.4		28.1	0.1	0.04	61.9	6.1	3.5
	No.5		35.7	7.7	3.1	56.8	1	2.4
^#^V2	No.1	0.38	42.1	14.1	5.6	71.1	14.5	0.4
	No.2		41.1	13.1	5.2	70	13.4	5.8
	No.3		37.9	9.9	4.0	72.1	15.5	5.4
	No.4		41.1	13.1	5.2	67.9	11.3	6.2
	No.5		40.0	12.0	4.8	62.6	6.1	4.5
^#^V3	No.1	0.41	37.1	9.1	3.6	65.6	9.4	2.4
	No.2		35.1	7.1	2.8	66.6	10.3	3.8
	No.3		39.0	11.0	4.4	64.6	8.4	4.1
	No.4		38.0	10.0	4.0	66.6	10.3	3.4
	No.5		37.1	9.1	3.6	63.6	7.4	4.1
^#^V4	No.1	0.39	33.8	5.8	2.3	61.2	4.5	3.0
	No.2		30.8	2.8	1.1	64.3	7.6	1.8
	No.3		34.9	6.9	2.8	57.0	0.4	3.0
	No.4		32.8	4.8	1.9	63.3	6.5	0.2
	No.5		34.9	6.9	2.8	63.3	6.5	2.6

**FIGURE 6:**
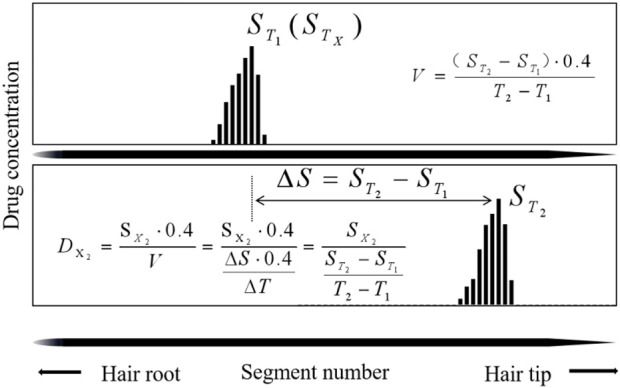
Calculation methods to estimate drug intake day ( $D_{X_{2}}$ ) by Temporal Intervals.

The second sampling time interval ( $D_{X_{2}}$ ) from the day of ingestion was estimated based on the formula of Eq.3, where $S_{X_{2}}$ represents the segment number that corresponds to the peak maxima of estazolam (Figure 6).

The second sampling time from the day of ingestion is estimated in Table 9. The deviations, which ranged from 0.2 to 15.5 days (average 7.4 ± 3.0 days, n = 20) between the calculated ( $D_{X_{2}}$ ) and actual day (56 days) calculated by “ $D_{X_{2}}$ -56 days”, included 20% within 2 days, 5% from 2 to 5 days, 45% from 5 to 10 days, and 30% beyond 10 days.

#### 4.2.2 The internal temporal marker (ITM) model

The ITMs are used to estimate the day of drug intake, based on extra drug ingestion and using the hair collected on 56 days: ${T^{\prime }}_{1}$ and ${T^{\prime }}_{2}$ represent the day of the event and the day of the second drug intake, respectively. $S_{{T^{\prime }}_{1}}$ and $S_{{T^{\prime }}_{2}}$ represent the segment numbers that correspond to the peak maxima of the drug for the first and the second drug administrations, respectively. The individual hair growth rate (V) was calculated using Eq. (4), based on the temporal intervals ( $\text{Δ}T^{\prime }$ ) between ${T^{\prime }}_{1}$ and ${T^{\prime }}_{2}$ , and the distance ( ΔS′ ) between $S_{{T^{\prime }}_{1}}$ and $S_{{T^{\prime }}_{2}}$ _._

$$V\prime =\frac{\text{Δ}S\prime \cdot 0.4}{\text{Δ}T\prime }=\frac{\left(S_{T\prime_{2}}-S_{T\prime_{1}}\right)\cdot 0.4}{T\prime_{2}-T\prime_{1}}$$ (4)

**FIGURE 7:**
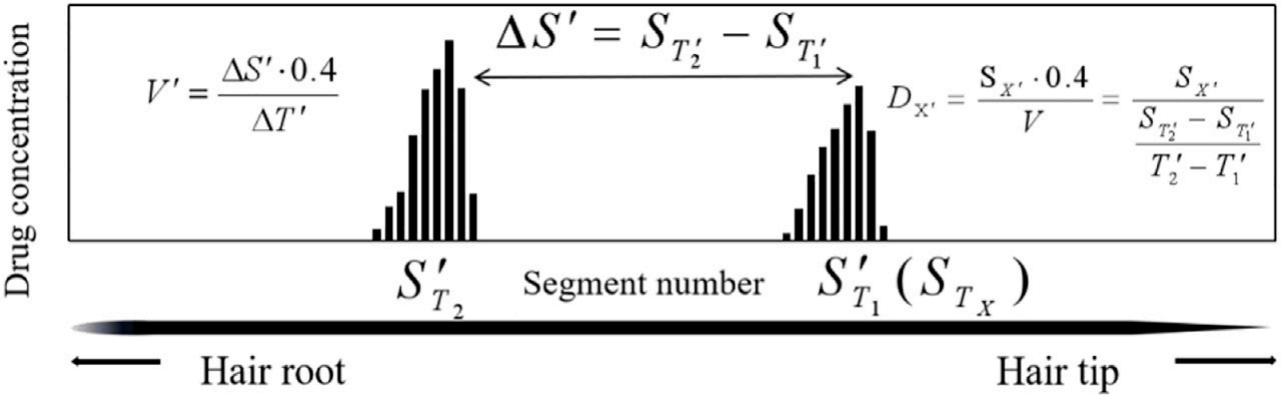
Calculation methods to estimate drug intake day ( $D_{X^{\prime }}$ ) by Internal Temporal Markers.

The first drug intake time ( $D_{X^{\prime }}$ ) was estimated based on the formula of Eq. (5), where $S_{X^{\prime }}$ represents the segment numbers that correspond to the peak maxima of estazolam (Figure 7).

$$D_{\text{X}\prime }=\frac{{\text{S}}_{X\prime }\cdot 0.4}{V}=\frac{{\text{S}}_{\text{X}\prime }\cdot 0.4}{\frac{\text{Δ}S\prime \cdot 0.4}{\text{Δ}T\prime }}=\frac{S_{X\prime }}{\frac{S_{T\prime_{2}}-S_{T\prime_{1}}}{T_{2}\prime -T\prime_{1}}}$$ (5)

The event time is estimated in Table 10. The deviations, which ranged from 0.5 to 9.6 days (average 4.3 ± 2.0 days,n = 20) between the calculated ( $D_{X^{\prime }}$ ) and actual day (56 days) calculated by “ $D_{X^{\prime }}$ - 56 days”, included 20% within 2 days, 50% from 2 to 5 days, and 30% from 5 to 10 days (Table 10).

**TABLE 10:** The estimation using ITMs (28ays and 56 days).

Voluntaries	Samples	Rate (mm/day)	Dx (Day)	Deviation	
				(Day)	(mm)
^#^V1	No.1	0.51	51.3	−4.7	1.9
	No.2	0.44	59.0	3	1.2
	No.3	0.53	57.5	1.5	0.6
	No.4	0.50	58.4	2.4	1.0
	No.5	0.44	60.5	4.5	1.8
^#^V2	No.1	0.41	64.6	8.6	3.4
	No.2	0.44	59.6	3.6	1.4
	No.3	0.41	65.6	9.6	3.8
	No.4	0.43	59.7	3.7	1.5
	No.5	0.39	61.1	5.1	2.0
^#^V3	No.1	0.46	58.4	2.4	1.0
	No.2	0.53	51.4	−4.6	1.8
	No.3	0.43	61.3	5.3	2.1
	No.4	0.43	63.4	7.4	3.0
	No.5	0.40	65.0	9	3.6
^#^V4	No.1	0.41	57.6	1.6	0.6
	No.2	0.43	57.7	1.7	0.7
	No.3	0.36	59.5	3.5	1.4
	No.4	0.41	55.5	−0.5	0.2
	No.5	0.44	59.5	3.5	1.4

### 4.3 Accuracy of the drug intake day estimation

The growth rate of individual hair strands was calculated using the ITM model and the temporal intervals model. Individual growth rates of ^#^V1 calculated by the two models in this study were 0.48 mm/day (n = 5) and 0.47 mm/day (n = 5), respectively, which were faster rates than determined for the other three volunteers. The hair strands were obtained from the same head region at the same time, but their growth rates differed considerably. Individual hair growth rates of 0.33–0.43 mm/day were calculated in the Shima study (Shima et al., 2019) and rates of 0.281–0.428 mm/day were calculated from nine victims of DFSA cases in the Wiedfeld study (Wiedfeld et al., 2021). In the present study (Shen and Xiang, 2020), analysis of the black hair samples revealed thicker hair follicles than the white hair samples, and the hair growth rate was faster. Hair growth is a cyclical process driven by changes in the activity of cytokines (hormones), which cause individual hairs on the body to be at various stages of the growth cycle (Kintz et al., 2015).

In this study, application of the individual growth rate calculated by Eq.1) and (4) after plucking the hair strands led to a gap of 1.7 ± 0.8 mm between the calculated and actual position for the ITM model and 3.0 ± 1.2 mm for the TI model. The two models were established based on a two-point hypothesis. First, the highest concentration segments were considered to correspond to the time of ingestion. Second, the individual hair growth rates were assumed to be constant over the study period. However, inconsistencies between theory and practice introduce errors into the time estimation.

Hair strands are normally cut near the scalp using scissors, or they are plucked out with the roots intact using tweezers. However, when cutting near the scalp, the actual length of the hair remaining on the scalp is unknown. In the Wiedfeld (Wiedfeld et al., 2021) study, the observed distance between the calculated and actual peak positions in the hair ranged between 2 and 7 mm (mean: 4.20 mm, median: 4.25 mm) for zolpidem. The explanation was as follows: the intradermal part of the hair from the dermal papilla to the scalp surface is approximately 4 mm, and 1–2 mm of hair remains on the surface after cutting the hair, resulting in a segment of 5–6 mm that is not calculated. The day of drug ingestion can be estimated more accurately using the distance from the hair root end (Kintz et al., 2015).

These findings can help further elucidate the drug incorporation mechanism, which is crucial for accurately interpreting hair analysis results revealing individual drug-use history.

## 5 Conclusion

The distribution of estazolam within a strand of hair was visualized based on micro-segmental analysis. We detected a significantly high concentration of estazolam in the hair bulb region, whereas a small peak was observed at the junction of the hair roots and shafts 12 h after administration, suggesting that the incorporation of estazolam occurred in two regions from the hair bulb, sweat, and/or sebum. TIMs and TIs were utilized to estimate the day of estazolam ingestion. The estimation accuracy between the calculated and actual drug peak position in hair was within an average error of 1.7 mm determined by the TIM model and 3.0 mm determined by the TI model.

## Data Availability

The original contributions presented in the study are included in the article/supplementary material, further inquiries can be directed to the corresponding author.
